# Influence of an Oxygen Carrier on the CH_4_ Reforming Reaction Linked to the Biomass Chemical Looping Gasification Process

**DOI:** 10.1021/acs.energyfuels.2c00705

**Published:** 2022-05-27

**Authors:** Iván Samprón, Luis F. de Diego, Francisco García-Labiano, María T. Izquierdo, Juan Adánez

**Affiliations:** Department of Energy and Environment, Instituto de Carboquímica−Consejo Superior de Investigaciones Científicas (ICB−CSIC)Miguel Luesma Castán 4, 50018 Zaragoza, Spain

## Abstract

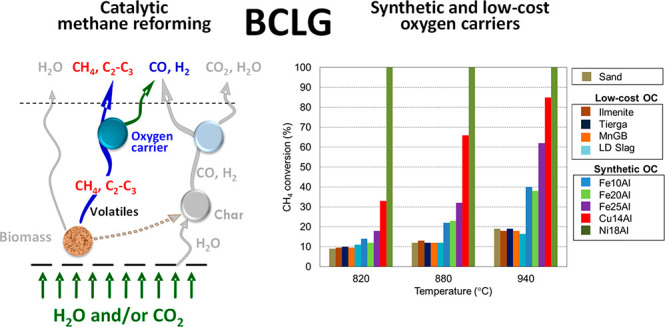

A major challenge in biomass chemical looping gasification (BCLG) is the conversion of CH_4_ and light hydrocarbons to syngas (CO + H_2_) when the goal is the use for bioliquid fuel production. In this work, tests were performed in a batch fluidized bed reactor to determine the catalytic effect on the CH_4_ reforming reaction of oxygen carriers used in the BCLG process. Three ores (ilmenite, MnGB, and Tierga), one waste (LD slag), and five synthetic materials (Fe10Al, Fe20Al, Fe25Al, Cu14Al, and Ni18Al) were analyzed. These results were compared to those obtained during ∼300 h of continuous biomass gasification operation in a 1.5 kW_th_ BCLG unit. The low-cost materials (ores and waste) did not show any catalytic effect in the CH_4_ reforming reaction, and as a consequence, the CH_4_ concentration values measured in the syngas produced in the continuous prototype were high. The synthetic oxygen carriers showed a catalytic effect in the CH_4_ reforming reaction, increasing this effect with increasing temperature. With the exception of the Ni-based oxygen carrier (used as a reference), the Cu-based oxygen carrier, working at 940 °C, showed the best catalytic properties, in good agreement with the low CH_4_ concentration values measured in the syngas generated in the continuous unit. The tests performed in a batch fluidized bed reactor were demonstrated to be very useful in determining the catalytic capacity of oxygen carriers in the CH_4_ reforming reaction. This fact is highly relevant when a syngas with a low CH_4_ content is desired as a final product.

## 1 Introduction

The International Energy Agency (IEA) Outlook of 2021 sets an increase of the biofuel demand of almost 3 times for 2050 in the net zero emission (NZE) scenario.^1^ The IEA indicates that the development of biofuels is a key issue for the decarbonization of the transport sector, especially for heavy trucks and aviation. Unfortunately, most of the biofuel demand is currently satisfied by conventional biofuels produced from food crop feedstocks, commonly referred to as first-generation biofuels, and include sugar cane ethanol, starch-based ethanol, fatty acid methyl ester (FAME), pure vegetable oil (SVO), and hydrotreated vegetable oil (HVO) produced from palm, rapeseed, or soybean oil.^1^ In contrast, IEA expects that 90% of the biofuel demand in 2050 NZE will be covered with advanced biofuels that do not compete with food for agricultural land and do not negatively affect sustainability.^1^

Thus, the production of syngas (CO and H_2_) through gasification processes from biomass forestry residues or agro-industrial wastes is an interesting way to produce advanced biofuels and other products. According to Sikarwar et al.,^2^ there are many routes already developed to transform the raw syngas into biofuels [Fischer–Tropsch (F–T) diesel or gasoline] or chemicals (ammonia, alcohols, ethanol, methanol, etc.), which could be directly used or converted into other chemicals [dimethyl ether (DME), methyl *tert*-butyl ether (MTBE), acetic acid, gasoline, etc.].

The main barrier of gasification processes is the energy source required for the endothermic gasification reactions. In one-step conventional gasification, energy could be supplied using an external energy input or burning part of the fuel. Gasification with air or oxygen has the disadvantages of producing a low-quality syngas by N_2_ dilution or the use of a costly air separation unit (ASU), respectively. Dual fluidized bed (DFB) gasification solves these problems using two interconnected fluidized beds. In this technology, heat required for endothermic gasification reactions is generated in a second reactor and transported by an inert solid (commonly silica sand) to the gasification reactor. The main problem of DFB gasification is that heat generation is carried out by the combustion of a part of the fuel (usually char), emitting CO_2_ to the atmosphere.

**Figure 1:**
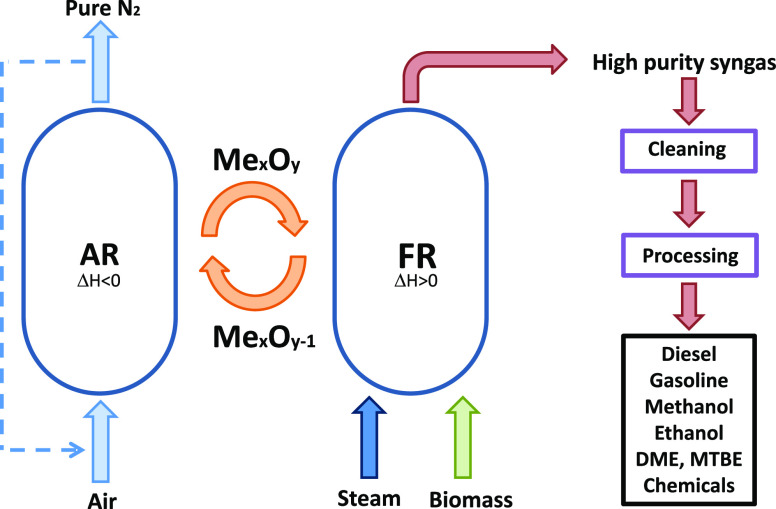
Scheme of the BCLG process.

In this sense, biomass chemical looping gasification (BCLG) is a novel technology that permits biomass gasification without an external power supply and, ideally, without CO_2_ emissions into the atmosphere. In BCLG, a solid oxygen carrier is used to transport oxygen and heat between two interconnected reactors (see Figure 1). In the fuel reactor (FR), the biomass is gasified and the oxygen carrier is reduced by its contact with the gases generated by devolatilization and gasification of the biomass. In the air reactor (AR), the oxygen carrier is regenerated with air, producing heat, owing to the exothermic nature of the oxygen carrier oxidation reaction. This heat is transported from the AR to the FR by the oxygen carrier, supplying the energy required for gasification reactions. Thus, a N_2_-free syngas stream is generated in the FR. Other advantages of BCLG over conventional gasification include lower tar production and reduced costs related to carbon capture because most of CO_2_ is generated in the FR. The FR outlet stream is sent to a cleaning step to remove impurities, such as tars, alkali, nitrogen compounds, and particulate matter (char and ash), because they could cause major problems in downstream processes. The syngas cleaning strategies used in this step are strongly related to the final applications of the syngas, and factors such as the nature of the biomass used should be integrated here.^3^

The selection of the oxygen carrier is one of the most studied topics in chemical looping processes. Although a wide range of oxygen carriers have been previously investigated for chemical looping combustion (CLC),^4−12^ the current challenge is to develop suitable oxygen carriers for BCLG, where lifetime is reduced with respect to CLC.^13,14^ In addition to lifetime, another challenge in BCLG that requires further study is the conversion of CH_4_ and light hydrocarbons to syngas to be used in F–T processes.

Several ores and wastes have been tested as oxygen carrier candidates in continuous operating CLG units as a result of their low costs. Lifetimes between 160 and 630 h have been reported.^13,15,16^ In addition, high amounts of CH_4_ (5–15 vol %) and light hydrocarbons, such as C_2_H_6_ and C_3_H_8_ (0.1–3.7 vol %), appeared in the syngas.^13,15−18^ Despite their higher costs, synthetic materials have also been proposed to improve some properties, such as lifetime.^14,19−21^ Our research group at ICB–CSIC has developed several Fe–Al oxygen carriers with the aim of extending their lifetime.^21^ Lifetime enhancement from 100 to 900 h was achieved by decreasing the Fe content from 25 to 10 wt % (quantified as Fe_2_O_3_). However, significant amounts of CH_4_ and lesser amounts of C_2_–C_3_ were observed in the syngas obtained under all experimental conditions and with all oxygen carriers tested.

Although C_1_–C_3_ compounds have high low heating values (LHVs) and are suitable when the syngas is used directly as a fuel (i.e., for a gas turbine), their conversion into H_2_ and CO is desirable for the production of liquid fuels (i.e., via F–T processes). Unfortunately, under typical gasification conditions, commonly used oxygen carriers are more reactive with H_2_ or CO than with CH_4_;^22^ therefore, the reduction of the oxygen carrier will probably take place with the oxidization of H_2_ and CO rather than CH_4_. This means that the best option to decrease the amount of CH_4_ in the syngas is to reform it with steam or CO_2_ to produce H_2_ and CO. A common way to reduce the amount of CH_4_ has been to increase the FR temperature.^14,17,19,21,23,24^ It is also well-known that some oxygen carriers have a catalytic effect on the CH_4_ reforming reaction. Ge et al.^18^ found that CH_4_ decreased from ∼8 to ∼4 vol % with an increasing hematite content from 40 to 60% on the bed material, a mix of hematite and silica sand. Similarly, our research group found a decrease in the CH_4_ content from 4.4 to 3.0 mol/kg of dry biomass fed, when the iron content in the oxygen carrier increased from 10 to 25%.^21^ Other authors added nickel to the oxygen carrier,^23,25^ as a result of its high reactivity with CH_4_, to increase syngas generation. Nonetheless, the use of nickel is not recommended here as a result of its high cost and toxicity.

In this work, the CH_4_ catalytic reforming capacity of different oxygen carriers was determined under typical BCLG conditions in a batch fluidized bed reactor, and the results were used to interpret the CH_4_ concentrations measured in a continuously operating BCLG unit in different operating conditions. The ultimate goal is to establish a simple experimental method to facilitate the selection of suitable oxygen carriers for continuously BCLG operating units without the need of conducting costly tests on continuous operating units.

## 2 Experimental Section

### 2.1 Oxygen Carriers

Nine oxygen carriers have been used in this work: three ores, one waste, and five synthetic materials. Eight of these materials have been previously used as oxygen carriers in a 1.5 kW_th_ continuous unit operating under BCLG conditions. Additionally, a synthetic oxygen carrier based on Ni, previously developed for CLC, has also been included. This oxygen carrier has been considered as a reference material as a result of its well-known catalytic properties with respect to CH_4_ reforming.

Low-cost materials included a Norwegian ilmenite from Titania A/S, a Spanish iron ore (Tierga), a Gabon manganese ore (MnGB), and a waste obtained in the steel industry (LD slag), which was supplied by SSAB Merox (Sweden). Synthetic materials were prepared by the incipient wetness impregnation method using alumina as a support and different metal oxides.^21,26^ These oxygen carriers were based on iron with different weight contents in metal oxide (Fe10Al, Fe20Al, and Fe25Al), copper (Cu14Al), and nickel (Ni18Al). Table 1 shows the main physical and chemical properties of oxygen carriers. The particle size of the oxygen carrier particles was determined in a Beckman Coulter LS13320 device. Density was measured in Micromeritics ACCUPYC II equipment. The porosity was determined by mercury porosimetry in a Micromeritics AUTOPORE V. The crushing strength was evaluated in a force gauge SHIMPO FGE-5X device. X-ray diffraction (XRD) analyses were carried out in a Bruker D8 Advance A25 diffractometer. Oxygen transport capacity, *R*_oc_, was obtained in a thermobalance (TGA CI Electronics) using a mixture of 15 vol % H_2_ + 20 vol % H_2_O as a reducing agent (N_2_ balance) and air for oxidation following the procedure described in ref (27).

**Table 1:** Main Physical and Chemical Properties of Fresh Oxygen Carriers

	ores			waste	synthetic				
	ilmenite	Tierga	MnGB	LD slag	Fe10Al	Fe20Al	Fe25Al	Cu14Al	Ni18Al
particle size (μm)	100–300	100–300	100–300	100–300	100–300	100–300	100–300	100–300	100–300
skeletal density (kg/m^3^)	4100	4216	3997	2764	3744	3950	4105	3699	2500
crushing strengh (N)	2.2	5.8	1.8	3.7	1.8	1.5	1.6	1.5	4.1
porosity (%)	1.2	26.3	35.7	14.1	50.2	45.6	44.4	50.0	42.5
oxygen transport capacity, *R*_oc_	0.043	0.077	0.056	0.018	0.01	0.02	0.025	0.029	0.038
main XRD phases	Fe_2_TiO_5_, Fe_2_O_3_, and TiO_2_	Fe_2_O_3_, SiO_2_, Al_2_O_3_, CaO, and MgO	Fe_2_O_3_, Mn_3_O_4_, and SiO_2_	CaO, Ca_2_SiO_4_, Ca_2_Fe_2_O_5_, Mg_2_Fe_2_Si_2_O_5_, CaMn_14_SiO_24_, Ca_3_Mg(SiO_4_)_2_, and Mg_2_SiO_4_	Fe_2_O_3_, α-Al_2_O_3_, and θ-Al_2_O_3_	Fe_2_O_3_, α-Al_2_O_3_, and θ-Al_2_O_3_	Fe_2_O_3_, α-Al_2_O_3_, and θ-Al_2_O_3_	CuO, CuAl_2_O_4_, α-Al_2_O_3_, and δ-Al_2_O_3_	NiO, NiAl_2_O_4_, and α-Al_2_O_3_

### 2.2 Batch Fluidized Bed Reactor

Tests to determine the catalytic effect of the oxygen carriers on methane conversion were carried out in a batch fluidized bed reactor facility described elsewhere.^28^ It consisted of a gas feeding system, a Kanthal-manufactured fluidized bed reactor, a two-way system to recover elutriated solids from the fluidized bed, and a gas analysis system. The gas feeding system had several mass flow controllers for specific gases. The fluidized bed reactor had an inner diameter of 0.054 m and 0.5 m height, with a 0.3 m preheat zone just below the distributor and was located inside an electrically heated furnace. The temperature in the fluidized bed was measured using a K-type thermocouple. A total of 300 g of each oxygen carrier was loaded in the fluidized bed reactor and heated under a N_2_ atmosphere. After the set temperature was reached, N_2_ was replaced by a synthetic gas (285 L_N_/h) composed of H_2_O, CO_2_, CO, and H_2_. When the oxygen carrier was reduced and the steady state was reached, a flow of 15 L_N_/h of CH_4_ was fed to obtain a syngas composition similar to that obtained during the BCLG operation (35 vol % H_2_O, 22 vol % CO_2_, 15 vol % CO, 23 vol % H_2_, and 5 vol % CH_4_). The gas composition obtained at the outlet of the batch reactor was continuously monitored in several online gas analyzers. The evolution of the CH_4_ concentration over time was determined in a Siemens Ultramat 23 analyzer. Several tests were performed with each of the oxygen carriers to determine the amount of CH_4_ converted over total CH_4_ fed at three different temperatures (820, 880, and 940 °C). The tests were repeated at least twice for each condition. Additional experiments were carried out at each temperature using sand as bed material to differentiate the catalytic effect of the oxygen carrier from the non-catalytic methane reforming reaction.

**Figure 2:**
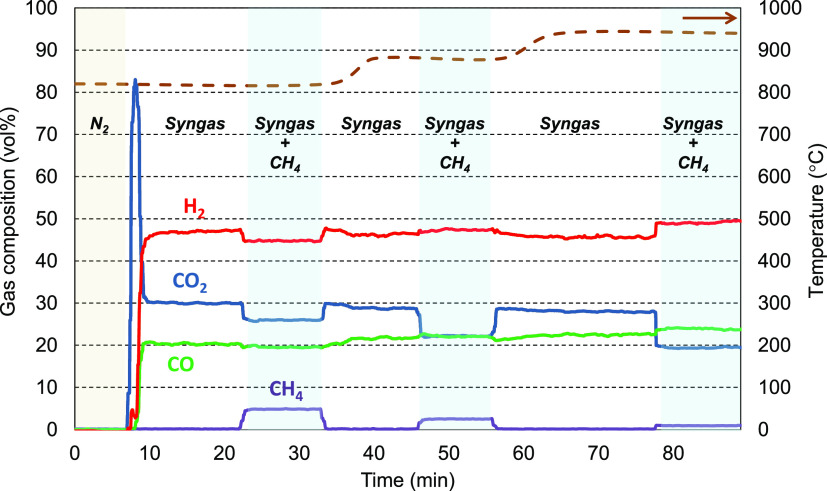
Typical gas composition profile for tests with the oxygen carrier Cu14Al.

Methane reforming includes both the reaction with steam and CO_2_, and the water–gas shift reaction was also considered.
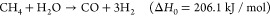
1
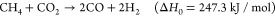
2
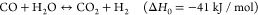
3An example of the flue gas composition profile (dry basis) is shown in Figure 2. During the first few minutes, no compounds were detected because N_2_ was the only gas introduced. After the introduction of the synthetic gas, the reducing gases (CO and H_2_) reacted with the oxygen carrier, generating a CO_2_ peak as a result of the oxidized state of the solid at the beginning. When the gas concentrations stabilized and steady state was reached, CH_4_ was fed. At that time, the CO_2_ concentration decreased as a consequence of the dilution caused by the addition of the 15 L_N_/h of CH_4_ and the CH_4_ dry reforming. In contrast, the dilution effect of CH_4_ on CO and H_2_ was partially offset by the generation of both gases caused by CH_4_ reforming. It has to be kept in mind that each mole of methane produces 4 mol of CO and H_2_ by reforming.

After the first test (performed at 820 °C), the flow of CH_4_ was stopped and the temperature was increased to the new set point (880 °C). When the temperature was reached and gas concentrations were stabilized, CH_4_ was fed again. In this case, lower CH_4_ concentrations were detected with respect to the previous test, indicating that the CH_4_ conversion was higher at 880 °C than at 820 °C. The same process was repeated at 940 °C, again observing an increase in the CH_4_ reforming reaction with increasing temperature.

The CH_4_ conversion, *X*_CH_4__ (%), was calculated as the molar flow of converted methane over the total methane fed (eq 1).
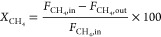
4In addition to the effect on the reforming reaction, the increase in the temperature also had an effect on the gas composition as a result of the water–gas shift reaction, shifting the equilibrium toward the production of CO and H_2_O and promoting a decrease in the CO_2_ and H_2_ concentrations. This effect was clearly seen in the gas compositions obtained when no CH_4_ was fed.

## 3 Results

Tests were performed in the batch fluidized bed reactor to determine the catalytic effect on the CH_4_ reforming reaction of oxygen carriers under typical conditions corresponding to BCLG operation. The results were compared to those obtained during continuous operation in a 1.5 kW_th_ BCLG unit located at ICB–CSIC. These data corresponded to ∼300 h of biomass gasification, where the effect of the main operation conditions, such as the FR temperature, oxygen/fuel ratio, λ, and steam/biomass ratio, S/B, was analyzed.

### 3.1 Material Characterization by XRD

The catalytic effect of the oxygen carrier on the CH_4_ reforming reaction depends upon not only the metal considered and its distribution over the internal surface of the oxygen carrier but also the oxidation state of the metal existing under BCLG conditions. This was the reason for carrying out the tests in the batch fluidized bed with a gas composition similar to that obtained in a BCLG process working in conditions close to autothermal operation. According to a previous work,^29^ this was achieved with oxygen/fuel ratios of about 0.3–0.35 for a steam/biomass ratio of 0.6 at different temperatures.

**Figure 3:**
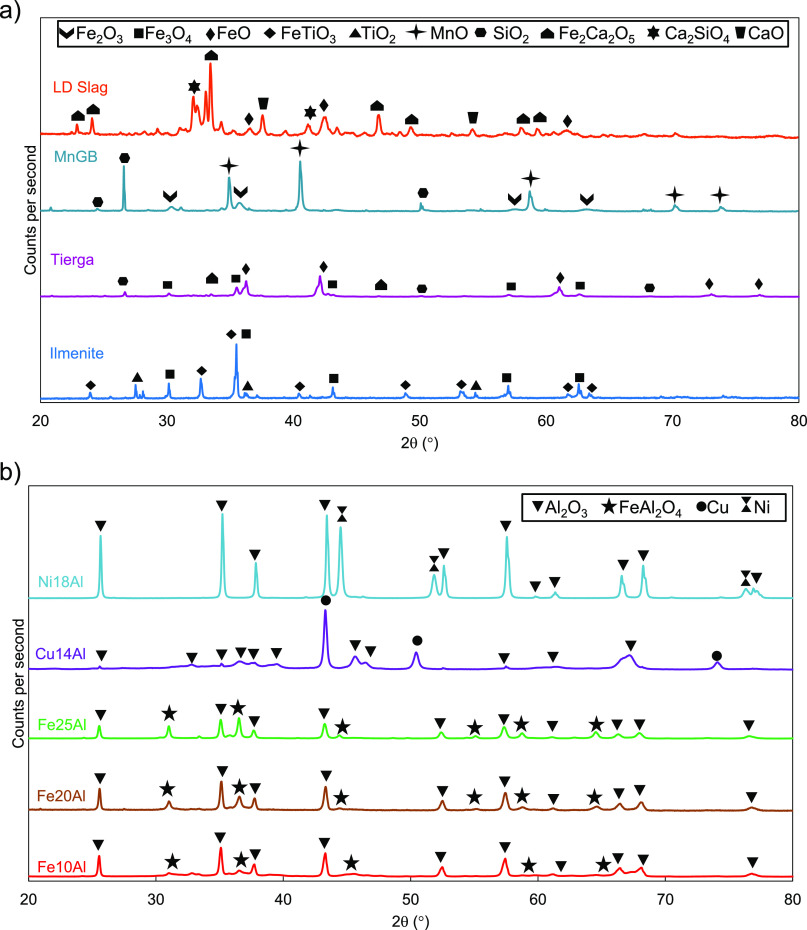
XRD profiles in reduced samples of the (a) low-cost materials and (b) synthetic oxygen carriers used in the batch fluidized bed.

Under the mentioned conditions, the oxygen carriers reached different reduced states depending upon thermodynamics, as seen in the XRD profiles shown in Figure 3, which correspond to reduced samples extracted from the reactor at the end of the tests. Table 2 shows the main reactions undergone by fresh oxygen carriers until reaching the reduced state. The reduction of the ilmenite ore led to the oxygen carrier to the reduced states FeTiO_3_ and Fe_3_O_4_. A major presence of Fe_3_O_4_ and FeO could be found for reduced Tierga, while MnO was the main reduced phase of MnGB. The identification of species by XRD was very complex for LD slag as a result of the high amount of compounds present in the oxygen carrier and the overlapping of peaks. Nonetheless, FeO was observed in the reduced sample.

**Table 2:** Main Reactions Undergone by Fresh Oxygen Carriers until Reaching the Reduced State

oxygen carrier	main reactions
ilmenite	Fe_2_TiO_5_ + TiO_2_ + H_2_/CO → 2FeTiO_3_ + H_2_O/CO_2_
	3Fe_2_O_3_ + H_2_/CO → 2Fe_3_O_4_ + H_2_O/CO_2_
Tierga	3Fe_2_O_3_ + H_2_/CO → 2Fe_3_O_4_ + H_2_O/CO_2_
	Fe_3_O_4_ + H_2_/CO → 3FeO + H_2_O/CO_2_
MnGB	Mn_3_O_4_ + H_2_/CO → 3MnO + H_2_O/CO_2_
LD slag	
FeXAl	Fe_2_O_3_·2Al_2_O_3_ + H_2_/CO → 2FeAl_2_O_4_ + H_2_O/CO_2_
Cu14Al	CuO + H_2_/CO → Cu + H_2_O/CO_2_
Ni18Al	NiO + H_2_/CO → Ni + H_2_O/CO_2_

Fe-based synthetic materials have been widely used as oxygen carriers as a result of their various oxidation states, Fe_2_O_3_–Fe_3_O_4_–FeO–Fe. Typically, the redox pair Fe_2_O_3_/Fe_3_O_4_ is considered for combustion, because further reduction states prevent complete use of the fuel. For this reason, Fe oxygen carriers are considered to have low oxygen transport capacities (*R*_o_ = 0.033). In gasification, the redox pair Fe_3_O_4_/FeO in addition to Fe_2_O_3_/Fe_3_O_4_ is also possible depending upon the operating conditions. However, synthetic Fe–Al oxygen carriers have the advantage of forming iron aluminate, FeAl_2_O_4_,^30^ regardless of operating conditions. Under typical gasification conditions, FeAl_2_O_4_ is the only stable phase of Fe–Al oxygen carriers, as seen in Figure 3b, and no further reduction to Fe is possible during BCLG operation. In contrast to Fe–Al oxygen carriers, metallic copper, Cu^0^, and metallic nickel, Ni^0^, are the reduced species of Cu14Al and Ni18Al materials under the above-mentioned conditions.

### 3.2 Study of the Oxygen Carrier Catalytic Effect

**Figure 4:**
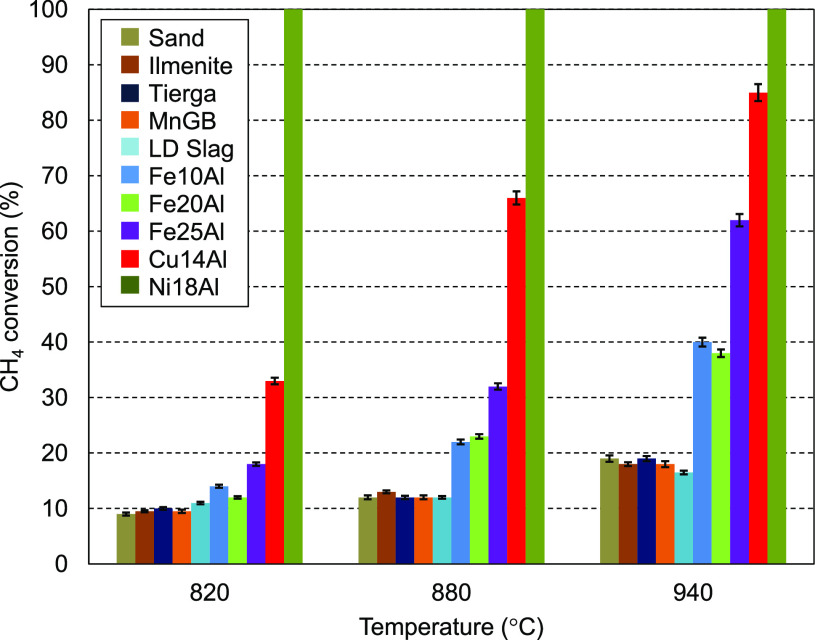
CH_4_ conversion in the batch fluidized bed reactor for the different oxygen carriers and temperatures.

Figure 4 shows the CH_4_ conversion obtained with the oxygen carriers at the three different temperatures. It was observed that the increase in the temperature had a positive effect on the CH_4_ conversion for all oxygen carriers. This means that the endothermic methane reforming reaction improved when more energy was supplied. Therefore, when the temperature increased, the equilibrium shifted toward the production of CO and H_2_.

The use of silica sand as bed material revealed that the non-catalytic methane reforming reaction produced CH_4_ conversion values from ∼10 to ∼18% as the temperature increased from 820 to 940 °C. CH_4_ conversions similar to the base case (silica sand) were obtained when the low-cost materials (ilmenite, Tierga, MnGB, and LD slag) were used, indicating that no catalytic effect occurred when using either ores or waste. In contrast, synthetic oxygen carriers exhibited higher CH_4_ conversions, especially at the highest temperature. As expected, the Ni-based oxygen carrier, Ni18Al, achieved complete methane conversion at any temperature, owing to the ability of Ni to catalyze hydrocarbon reforming reactions.^31^ From Rietveld refinement of the XRD pattern, 17.1 wt % of the crystal phase corresponds to Ni^0^, and it can be deduced that it is well-dispersed, because the crystal size is 56 nm. The Cu-based oxygen carrier, Cu14Al, showed good catalytic properties, reaching high CH_4_ conversion values, especially at 940 °C, at which 85% of CH_4_ was converted. A higher temperature could increase the catalytic effect of this oxygen carrier, but temperatures above 940 °C are not suitable as a result of agglomeration issues. This could be explained as a result of the reduction pathway of the oxygen carrier Cu14Al, which is always directly reduced from CuO to Cu^0^, as seen in the XRD profile (Figure 3b), and Cu^0^ was well-dispersed with a crystal size of 40 nm. It is well-known that metallic copper and metallic iron have a catalytic effect in different reactions.^32−34^

Although it was not thermodynamically possible to achieve metallic iron under the above conditions, the catalytic effect of the Fe25Al oxygen carrier was also important, reaching ∼60% of CH_4_ conversion at 940 °C. The other Fe-based synthetic materials with a lower Fe content, Fe10Al and Fe20Al, showed a lower catalytic effect (∼40% conversion of CH_4_ at 940 °C) than Fe25Al in the reforming reaction. The XRD analysis and further quantification of the Fe-based synthetic oxygen carriers, Fe10Al, Fe20Al, and Fe25Al, showed that the only Fe-containing crystal phase was FeAl_2_O_4_. No metallic iron was detected (by thermodynamic restrictions at BCLG conditions), and lower catalytic activity was expected as a result of this fact. The FeAl_2_O_4_ crystal phase content was 4.8, 10.6, and 43 wt % for Fe10Al, Fe20Al, and Fe25Al, respectively, with crystal sizes of 5, 15, and 23 nm, respectively. The CH_4_ conversion capacity was quite similar for both Fe10Al and Fe20Al oxygen carriers, with lower phase content, and significantly higher for the Fe25Al oxygen carrier, indicating that the possible catalytic effect of the FeAl_2_O_4_ phase would be more attributable to the phase content than to the dispersion. However, the CH_4_ conversion capacity was lower than that of Cu14Al, in which metallic copper was found.

Metallic Fe was never found for ilmenite and LD slag under the conditions mentioned above. Similarly, Tierga iron ore was reduced to Fe_3_O_4_ and FeO, under typical gasification conditions existing in BCLG processes (see Figure 3a). Further reduction to Fe^0^ would only be possible in the presence of a higher concentration of reducing gases, but these conditions are not possible in the BCLG autothermal operation. The non-formation of metallic Fe and the low activity of the crystal phases in the reduced samples as a result of the low dispersion as the crystal size being higher than 100 nm seem to be responsible for the fact that low-cost materials have a negligible catalytic effect in the CH_4_ reforming reaction.

### 3.3 CH_4_ Concentrations Measured in a 1.5 kW_th_ Unit Operating under BCLG Conditions

The ICB–CSIC research group tested the behavior of the aforementioned oxygen carriers, with the exception of the Ni-based oxygen carrier, during continuous operation in a 1.5 kW_th_ unit under BCLG conditions. The unit consisted of two bubbling interconnected fluidized beds, FR and AR, with the oxygen carrier circulating between them. The solid circulation rate was perfectly controlled by means of a solid valve. Biomass was fed to the FR by means of a double screw-feeder system. The reduced solids from the FR were sent to the AR where they were oxidized and returned to the FR to start a new cycle. A more detailed description of the installation can be found elsewhere.^35^

Main operating parameters, such as the oxygen/fuel ratio, steam/biomass ratio, and temperature, were studied for each oxygen carrier. With each solid, about 50 h of hot solid circulation and 35 h of biomass gasification were performed. The lattice oxygen provided by the oxygen carrier to produce syngas in the FR was controlled by limiting the air feed in the AR. This method allowed for a smooth operation, keeping the fluid dynamic properties of the system constant under different operating conditions. A more detailed description of operation can be found elsewhere.^13,14^

The conclusions derived from those tests on the effect of operating conditions on CH_4_ conversion were the following: (1) Methane appeared at the FR outlet in all operating conditions and for all oxygen carriers and biomasses used. Methane concentration values from 3 to 10 vol % (dry basis) were found. This fact has also been observed by other authors who operated BCLG continuous units, independent the unit and method used to control the oxygen/biomass ratio.^13−18^ (2) The oxygen/biomass and the steam/biomass ratios slightly affected the CH_4_ concentration obtained at the FR outlet for a given oxygen carrier.^13^ (3) The oxygen carrier was the issue that most affected the CH_4_ concentration, in addition to the operating temperature. A summary of the experiments carried out in the 1.5 kW_th_ BCLG unit is shown in Table 3.

**Table 3:** Summary of BCLG Tests Performed in the 1.5 kW_th_ Unit, S/B ∼ 0.6

			gas composition (mol/kg of dry biomass)					
	temperature (°C)	λ	CO_2_	CO	H_2_	CH_4_	C_2_–C_3_	reference
ilmenite	820	0.3–0.4	16.9–20.0	8.9–9.9	11.7–11.8	5.4–6.6	1.8–1.9	(13)
	880	0.2–0.3	16.2–22.2	10.2–16.0	13.8–23.0	5.5–6.2	1.1–1.5	
	940	0.2–0.3	16.9–21.7	11.5–16.9	13.1–23.9	5.4–6.0	1.1–1.8	
Tierga	820	0.3–0.5	18.6–25.0	7.5–10.8	8.4–14.8	4.4–5.4	0.9–1.2	(15)
	880	0.3–0.4	23.2–25.6	8.2–10.2	11.5–14.8	4.9–5.7	0.8–1.0	
	940	0.2–0.5	18.7–28.7	8.3–15	9.0–20.4	4.5–5.3	0.8–1.0	
MnGB	820	0.2–0.4	13.8–22.3	8.2–11.3	11.1–18.9	4.3–4.7	1.2–1.3	(15)
	880	0.3–0.4	20.0–23.8	9.7–12.2	12.1–17.8	4.4–5.1	0.9–1.0	
	930	0.2–0.4	16.9–23.7	11.4–15.6	10.8–23.0	4.7–5.0	0.6–1.0	
LD slag	820	0.2–0.4	16.7–22.3	9.3–13.0	15.0–23.2	4.3–4.7	1.2–1.4	(16)
	880	0.2–0.5	16.9–23.8	10.0–16.0	12.9–25.4	4.4–5.1	0.3–1.0	
	930	0.2–0.4	15.4–25.5	11.9–20.3	10.0–26.7	4.7–5.3	0.3–1.1	
Fe10Al	820	0.2	18.7	10.1	21.4	4.5	0.4	(21)
	880	0.3	20.4	11.3	20.8	4.3	0.4	
	940	0.2–0.4	16.3–24.1	11.6–16.4	16.5–26.8	3.9–4.9	0.1–0.3	
Fe20Al	820	0.3–0.5	18.8–24.1	6.2–9.0	13.7–21.9	4.0–4.5	0.8–0.9	(14)
	880	0.2–0.4	16.6–24.2	10.4–12	18.5–24.5	4.5–4.9	0.5–0.6	
	940	0.2–0.6	16.3–30.9	7.7–15.6	12.3–26.5	3.9–4.8	0.2–0.4	
Fe25Al	820	0.3	19.5	11.2	22.9	3.6	0.5	(21)
	880	0.3	20.4	14.9	25.5	3.5	0.2	
	940	0.2–0.4	16.0–22.9	14.7–18.1	20.9–28.5	2.6–3.2	0.1	
Cu14Al	820	0.3	12.6	12.3	27.4	4.0	0.4	article in preparation
	880	0.3	14.1	14.5	27.6	3.2	0.3	
	940	0.3–0.4	17.1–18.0	17.7–19.6	25.9–31.3	1.9–2.3	0	
Ni18Al	820	0.3	28.7	23.1	47.2	0.9	0	this work
	880	0.3	28.5	24.2	46.1	0.8	0	
	940	0.3	28.3	25.9	45.1	0.7	0	

Although Ni is toxic and should not be used with solid fuels, three tests with the Ni-based oxygen carrier were performed for comparison purposes in the continuous unit. The operating conditions were similar to those previously used with the other oxygen carriers. The main results are included in Table 3.

**Figure 5:**
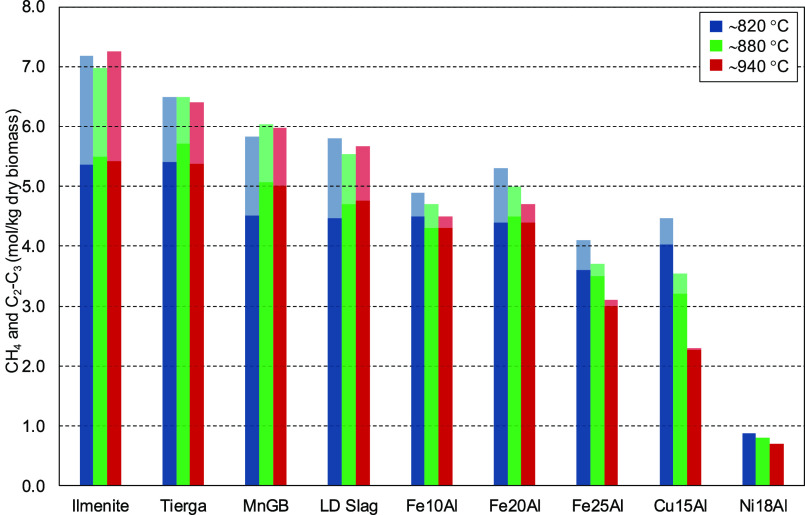
Comparison of CH_4_ (dark color) and C_2_–C_3_ (light color) contents in the syngas obtained in the 1.5 kW_th_ unit for the different oxygen carriers (S/B ∼ 0.6, and λ ∼ 0.3).

Figure 5 shows the CH_4_ and C_2_–C_3_ concentrations (in moles per kilogram of dry biomass) obtained for the different oxygen carriers at three temperatures (820, 880, and 930–940 °C), an oxygen/fuel ratio, λ, of ∼0.3, and a steam/biomass ratio, S/B, of ∼0.6.

At ∼820 °C, high amounts of CH_4_ were obtained for most of the oxygen carriers. The ilmenite and Tierga iron ore produced the highest CH_4_ concentrations, reaching values above 5.0 mol/kg of dry biomass. When the temperature increased, two different behaviors were found. The CH_4_ concentration remained constant with the temperature for low-cost oxygen carriers (ilmenite, Tierga, MnGB, and LD slag) and Fe10Al and Fe20Al synthetic oxygen carriers. On the contrary, the CH_4_ concentration decreased for the Fe25Al and Cu14Al synthetic oxygen carriers. Excluding the Ni18Al oxygen carrier, the lowest values of the CH_4_ concentration were obtained using Cu14Al, which exhibited a decrease in the CH_4_ content from 4.0 mol/kg of dry biomass at 820 °C to 2.3 mol/kg of dry biomass at 940 °C.

It is noteworthy that some CH_4_ appeared when Ni18Al was used as an oxygen carrier, even at the highest temperature of 940 °C, with values of ∼0.8 mol/kg of dry biomass. This result is remarkable considering the good catalytic properties of this material, as confirmed by the tests carried out in the batch fluidized bed, where a complete conversion of CH_4_ was obtained at the three temperatures tested. The presence of CH_4_ at the FR outlet would indicate that not only are the catalytic properties of the material used as the oxygen carrier relevant but also the hydrodynamic conditions existing in the reactor. In this case, a poor contact between the oxygen carrier and part of CH_4_ generated during biomass devolatization could occur during the experiments carried out in the continuous unit. Therefore, all of the methane concentrations obtained in a continuous operation unit could be reduced if the design of the FR is adapted to improve the contact between the oxygen carrier and the gases generated during biomass gasification. In fact, there are already innovative proposals developed to improve the solid–gas contact in the FR.^36^

In the same way as for CH_4_, light hydrocarbons were generated in different amounts depending upon the gasification conditions. In fact, the amounts of C_2_H_6_ and C_3_H_8_ were clearly related to the CH_4_ content, with C_2_H_6_ and C_3_H_8_ being converted into other compounds (CO, H_2_, CH_4_, etc.) when CH_4_ was also converted into CO and H_2_.

**Figure 6:**
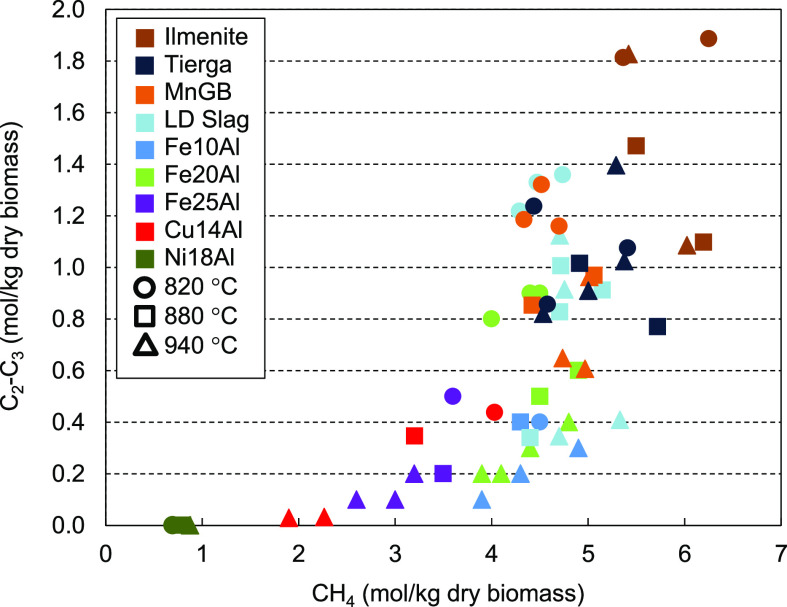
Relationship between the amounts of CH_4_ and C_2_–C_3_ generated during continuous operation in the 1.5 kW_th_ BCLG unit (λ ∼ 0.3, S/B ∼ 0.6, and *T* = 820–940 °C).

Figure 6 shows the relationship between the amount of light hydrocarbons and CH_4_ produced in the gasification tests performed in the 1.5 kW_th_ unit. As observed, when low values of CH_4_ were obtained (0.7–3.5 mol/kg of dry biomass), low contents of C_2_–C_3_ also appeared [<0.5 mol/kg dry biomass (db)]. In contrast, when CH_4_ was hardly reformed (values up to ∼4.0 mol/kg db), light hydrocarbons appeared in a wide range of quantities (0.6–1.9 mol/kg db), with their reforming being conditioned to the rest of operating parameters, such as the temperature. Thus, when oxygen carriers with a high catalytic effect on CH_4_ reforming were used, i.e., Cu14Al, Fe25Al, and Ni18Al, low values of light hydrocarbons were obtained. Meanwhile, with oxygen carriers with little or no effect on CH_4_ reforming, as was the case with low-cost materials, the amounts of C_2_ and C_3_ were greater and their reforming depended more upon the operating conditions.

### 3.4 Comparison of the Results Obtained in the Batch Reactor and in the 1.5 kW_th_ Unit

**Figure 7:**
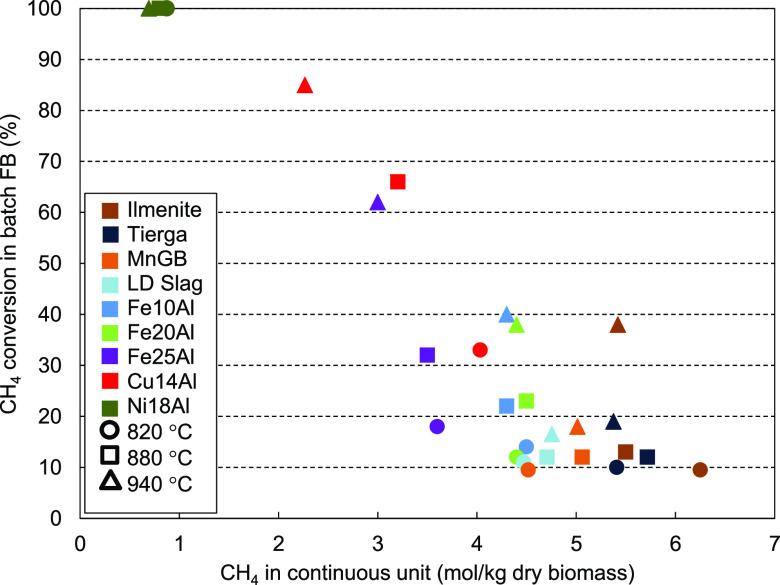
Relationship between CH_4_ conversion obtained in batch tests and CH_4_ content obtained during operation in the 1.5 kW_th_ continuous unit (S/B ∼ 0.6, λ ∼ 0.3, and *T* = 820–940 °C).

Excluding the effect of hydrodynamic conditions affecting the CH_4_ conversion during operation in a continuous unit, it seems clear that the catalytic effect of oxygen carriers was the major issue affecting the CH_4_ content of the syngas. Figure 7 shows the relationship between the CH_4_ conversion obtained in the batch fluidized bed tests with the different oxygen carriers and the CH_4_ concentration obtained during operation in the 1.5 kW_th_ BCLG unit.

It was observed that the oxygen carriers exhibiting high catalytic capacity in the batch fluidized bed reactor (Ni18Al, Cu14Al, and Fe25Al) were also the oxygen carriers that presented the lowest concentrations of CH_4_ in the continuous unit. Furthermore, an increase in the temperature promoted the catalytic effect of these oxygen carriers, as seen in Figure 7. The remaining oxygen carriers, except for Fe20Al and Fe10Al, which showed some catalytic effect at the highest temperatures tested, were not good catalysts for the CH_4_ reforming reaction. These oxygen carriers acted only as inert bed material in both the batch fluidized bed and the continuous unit, and therefore, high concentrations of CH_4_ were obtained in the continuous operating unit.

In conclusion, although the results obtained may be slightly affected by the behavior of the reactor as a result of variations in the solid–gas contact, tests in a batch fluidized bed reactor can be very useful to determine the catalytic capacity of an oxygen carrier in the CH_4_ reforming reaction, avoiding the need to perform costly tests on continuously operating units. This is important when syngas with a very low CH_4_ content is needed, as is the case of the syngas used for the production of liquid fuels by the F–T process.

## 4 Conclusion

The CH_4_ catalytic reforming capacity of different oxygen carriers was tested under typical BCLG conditions in a batch fluidized bed reactor, and the results obtained were used to interpret the CH_4_ concentrations measured in a continuous BCLG prototype working under different operating conditions. Three ores (ilmenite, MnGB, and Tierga), a waste (LD slag), and five synthetic materials (Fe10Al, Fe20Al, Fe25Al, Cu15Al, and Ni18Al) were analyzed. The following was found: (1) The low-cost materials (ores and waste) did not show any catalytic effect on the CH_4_ reforming reaction, and as a consequence, the CH_4_ concentration values measured in the syngas produced in the continuous prototype were high. (2) The synthetic oxygen carriers showed a catalytic effect in the CH_4_ reforming reaction, increasing this effect with an increasing temperature. The catalytic effect was low with the Fe10Al and the Fe20Al oxygen carriers and improved with the Fe25Al oxygen carrier. With the exception of the Ni-based oxygen carrier (used as a reference), the Cu-based oxygen carrier, Cu14Al, showed the best catalytic properties, reaching high CH_4_ conversion values, especially at 940 °C, where 85% of CH_4_ was converted to CO and H_2_. With this oxygen carrier, low values of the CH_4_ concentration were measured in the syngas generated in the continuous unit, especially when the unit operated at 940 °C. (3) Tests in a batch fluidized bed reactor have been demonstrated to be very useful to determine the catalytic capacity of an oxygen carrier in the CH_4_ reforming reaction. Knowledge of this catalytic capacity provides information on great relevance to estimate the amount of CH_4_ in the syngas generated during the operation of pilot plants, without the need to carry out expensive experimental tests in these units. This fact is important when syngas with a very low CH_4_ content is needed, as is the case with the syngas used for the production of liquid fuels by the F–T process.
